# Possible mechanisms to improve sleep spindles via closed loop stimulation during slow wave sleep: A computational study

**DOI:** 10.1371/journal.pone.0306218

**Published:** 2024-06-26

**Authors:** Muhammad Mushtaq, Lisa Marshall, Rizwan ul Haq, Thomas Martinetz

**Affiliations:** 1 Institute for Neuro- and Bioinformatics, Lübeck, Germany; 2 Institute of Experimental and Clinical Pharmacology, University of Lübeck, Lübeck, Germany; 3 Center of Brain, Behavior and Metabolism, Lübeck, Germany; 4 University Clinic Hospital Schleswig Holstein, Lübeck, Germany; 5 Department of Pharmacy, Abbottabad University of Science and Technology, Abbottabad, Pakistan; University of Nebraska Medical Center College of Medicine, UNITED STATES

## Abstract

Sleep spindles are one of the prominent EEG oscillatory rhythms of non-rapid eye movement sleep. In the memory consolidation, these oscillations have an important role in the processes of long-term potentiation and synaptic plasticity. Moreover, the activity (spindle density and/or sigma power) of spindles has a linear association with learning performance in different paradigms. According to the experimental observations, the sleep spindle activity can be improved by closed loop acoustic stimulations (CLAS) which eventually improve memory performance. To examine the effects of CLAS on spindles, we propose a biophysical thalamocortical model for slow oscillations (SOs) and sleep spindles. In addition, closed loop stimulation protocols are applied on a thalamic network. Our model results show that the power of spindles is increased when stimulation cues are applied at the commencing of an SO Down-to-Up-state transition, but that activity gradually decreases when cues are applied with an increased time delay from this SO phase. Conversely, stimulation is not effective when cues are applied during the transition of an Up-to-Down-state. Furthermore, our model suggests that a strong inhibitory input from the reticular (RE) layer to the thalamocortical (TC) layer in the thalamic network shifts leads to an emergence of spindle activity at the Up-to-Down-state transition (rather than at Down-to-Up-state transition), and the spindle frequency is also reduced (8–11 Hz) by thalamic inhibition.

## Introduction

Slow oscillations (SOs) and sleep spindles are two characteristic EEG rhythms of non-rapid eye movement (NREM) sleep, and are attributed essential roles in memory consolidation [1–4]. According to the concept of active system consolidation, cortical SOs provide a temporal window for neuronal reactivation processes during which the transfer of recently acquired memory traces to the cortex for long-term storage is presumed to occur [5–8]. Burst-like waxing and waning EEG sleep spindles are the hallmark of NREM sleep stage N2, but are also found in N3,often concurrent with SOs in a phase dependent manner [9–12]. Hippocampal sharp wave ripples (SWRs) present a further electrophysiological event occurring phase-dependently to SOs, and also spindles, and involved in memory consolidation [13]. The simultaneous occurrence of these electrophysiological events reflecting parallel neuronal network activity is indicative of inter-regional communication and cellular plasticity such as long-term potentiation [14–18]. Long-term potentiation like processes were found to specifically occur during sleep spindle-like activity [19]. Combined behavioral and EEG studies in humans and experimental animals show an association between memory consolidation and spindle activity (e.g., spindle density or/and sigma power) [15, 17, 20–22].

Several experimental studies show that spindle activity can be enhanced through sensory or electrical stimulation during sleep [23, 24]. Closed-loop acoustic stimulation (CLAS), i.e., acoustic stimulation applied during a specific phase of the SO, presents an intriguing method to study network and behavioral responsiveness in a phase-dependent manner, even across species. CLAS is able to modify endogenous slow oscillations, sleep spindles, and hippocampal sharp-wave ripple activity [25–30] and has been shown to enhance sleep-associated memory consolidation [25, 29, 31]. For the response to CLAS brain state and timing (e.g., stimulation phase of the oscillatory rhythm) have proven decisive [28, 30, 32]. CLAS is often used as a double stimulation with two stimuli given at the same phase of two successive SOs. Yet, some CLAS protocols employed single or trains of stimuli delivered phase dependently [26, 31]. In humans, CLAS presents a noninvasive tool to potentially improve sleep functions and is more versatile than targeted memory reactivation (TMR) [33].

In this study, we present a thalamocortical computational model for NREM sleep that produces fast spindles along with the SO rhythm [34, 35], and most importantly generates the response of fast sleep spindles to stimulation cues. Several closed loop stimulation protocols are implemented. Stimulation cues are given to the model by introducing a sensory neuron (SN) that targets the TC layer of the thalamic network via AMPA receptors. The purpose of this study is to develop a computational model, that can closely simulate experimental results of closed loop acoustic stimulation, and can thus subsequently be used to test the efficacy of closed loop stimulation protocols during NREM sleep to enhance sleep spindle activity. SO states for stimulation cues for both double-click closed loop stimulations (CLS), single-click closed loop stimulations (sCLS), and driving stimulations (DSt) are precisely detected by the model. To better simulate acoustic stimuli, the computational current injection targets the thalamic network via a SN.

## Methods

**Fig 1:**
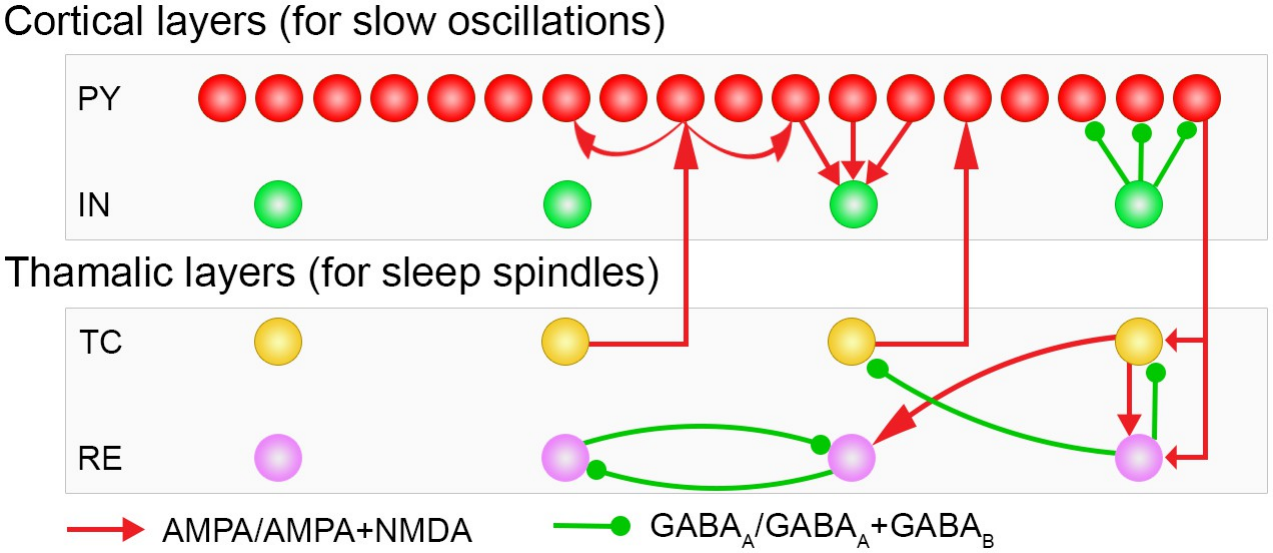
The thalamocortical network geometry. The network is comprised of four cell layers. The top two consist of cortical PY and IN cells. The bottom two, thalamic TC and RE neuron layers, generate sleep spindles. The cortical PY cells layer contains 200 neurons, all other layers contain 40 neurons. Small, green-filled circles symbolize locations of GABA_A_Rs or GABA_A_Rs+ GABA_B_Rs, and the green lines corresponding afferent connections. Red arrowheads point, to AMPARs or AMPARs+ NMDARs receptors, with the red lines indicating the corresponding connections.

Our conductance-based thalamocortical model for NREM sleep computes intrinsic and synaptic currents for all network neurons. The intrinsic currents and their conductances are described in Tables 1 and 2, the synaptic currents in Table 3. The thalamocortical network model consists of two interacting sub-networks (Fig 1). The first one is a two-layer cortical network for SOs generation comprised of pyramidal (PY) and interneuron (IN) cells. At first, the dynamics of cortical cells in the model were fitted to animal data obtained from Wistar rats from layer V of the neocortical brain slice [36]. The second thalamic network is also comprised of two cell layers: thalamocortical/relay (TC) and reticular (RE) cells and generates sleep spindles. The thalamic sleep spindles are initiated by the interaction of hyperpolarization current (I_*h*_) and the transient calcium current (I_*T*_) of TC and RE cells [37, 38]. The dynamics of intrinsic currents of the thalamic cells were fitted to data obtained from rats and guinea pig [39, 40]. Thalamic and cortical network neurons are modeled by Hodgkin-Huxley kinetics.

**Table 1:** Dynamical models of all Intrinsic currents.

Type of current	Dynamic of current
**For PY cells**	
(Axosomatic + dendritic) Fast sodium current *I*_Na_	
	*M* = 3; *H* = 1
	α1 = 0.182(*V* + 25)/(1 − exp(−(*V* + 25)/9)) *if* |*V* − 10|/35 > 10^−6^
	= 1.638 *if* |*V* − 10|/35 < 10−6
	β1 = 0.124(−(*V* + 25))/(1 − exp((*V* + 25)/9)) *if* |*V* − 10|/35 > 10^−6^
	= 1.116 *if* |*V* − 10|/35 < 10^−6^
	τm = (1/(α1 + β1))/2.9529;
	*m*∞ = α1/(α1 + β1);
	α2 = 0.024(*V* + 40)/(1 − exp(−(*V* + 40)/5)) *if* |*V* − 10|/50 > 10^−6^
	= 0.12 *if*|*V* − 10|/50 < 10^−6^
	β2 = 0.0091(*V* − 85)/(1 − exp(−(*V* − 85)/5)) *if* |*V* − 10|/50 > 10^−6^
	= 0.0455 *if* |*V* − 10|/50 > 10^−6^
	τh = (1/(α2 + β2))/2.9529;
	*h*∞ = 1/(1 + exp((*V* + 55)/6.2));
(Axosomatic & dendritic) Fast potassium current *I*_K_	
	*M* = 0;*N* = 1;
	α = 0.02 * (*V* − 25)/(1 − exp(−(*V* − 25)/9));
	β = −0.002 * (*V* − 25)/(1 − exp((*V* − 25)/9));
	τ*n* = (1/(α + β))/2.9529;
	*n*∞ = α/(α + β);
(Axosomatic + dendritic) Persistent sodium current *I*_Na(p)_	
	*M* = 1;*N* = 0;
	*m*∞ = 0.2/(1 + *exp*(−(*v* + 42)/5));
(Dendrite) Slow voltage-dependent non-inactivating potassium current *I*_Km_	
	*M* = 1; *N* = 0;
	α = 0.001 × (*V* + 30)/(1 − exp(−(*V* + 30)/9));
	β = −0.001 × (*V* + 30)/(1 − exp((*V* + 30)/9));
	τm = (1/(α + β))/2.9529;
	*m*∞ = α/(α + β);
(Dendrite) Slow calcium-dependent potassium current *I*_KCa_	
	*M* = 1; *N* = 0;
	α = 0.01 × [Ca^2+^]*i*;
	β = 0.02;
	τm = (1/(α + β))/2.9529;
	*m*∞ = α/(α + β);
(Dendrite) High threshold calcium current *I*_HVA_	
	*M* = 2; *N* = 1;
	α1 = 0.055 × (−27 − *V*)/(exp((−27 − *V*)/3.8) − 1);
	β1 = 0.94 × exp((−75 − *V*)/17);
	τm = (1/(α1 + β1))/2.9529;
	*m*∞ = α1/(α1 + β1);
	α2 = 0.000457 × exp((−13 − *V*)/50);
	β2 = 0.0065/(exp((−*V* − 15)/28) + 1);
	τh = (1/(α2 + β2))/2.9529;
	*h*∞ = α2/(α2 + β2);
(Dendritic) Potassium leak current *I*_KL_	
	*M* = 0; *N* = 0;
	*g*KL = 0.0033*mS*/*cm*^*2*^;
**For IN cells**	
	IN cells have the same current dynamics as PY cells except *I*_NA(p)_. *I*_NA(p)_ is not included in IN cells
**For TC cells**	
Fast sodium current *I*_Na_	
	*M* = 3; *H* = 1;
	α1 = 0.32 × (−37 − *v*)/(exp((13 − (*V* + 40))/4) − 1);
	β1 = 0.28 × (*V* − 90)/(exp(((*V* + 40) − 40)/5) − 1);
	*m*∞ = α1/(α1 + β1);
	τm = 1/(α1 + β1);
	α2 = 0.128 × exp((17 − (*V* + 40))/18);
	β2 = 4/(exp((40 − (*V* + 40))/5));
	*h*∞ = α2/(α2 + β2);
	τh = (α2 + β2);
Fast potassium current *I*_K_	
	*M* = 0; *N* = 4;
	α1 = 0.032 × (−35 − *V*)/(exp((−35 − *V*)/5) − 1);
	β1 = 0.5 × exp((−40 − *V*)/40);
	n∞ = α1/(α1 + β1);
	τn = 1/(α1 + β1);
Hperpolarization-activated cation current *I*_h_	
	*Voltage dependence*: $C\overset{\alpha }{\to }O,\;O\overset{\beta }{\to }C$
	*h*∞ = 1/(1 + exp((*V* + 75)/5.5));
	τs = (20 + 1000/(exp((*V* + 71.5)/14.2) + exp(−(*V* + 89)/11.6)));
	α = *h*∞/τs
	β = (1 − *h*∞)/τs
	*Calcium dependence*:
	$P0+2Ca\overset{k1}{\to }P1,\;\;\;P0+2\overset{k2}{\to }Ca;\;\;O+P1\overset{k3}{\to }OL,\;\;OL\overset{k4}{\to }O+P1$
	*k*1 = 2.5×107*mM*^-4^, *k*2 = 4×10^−4^ *ms*^*-1*^, *k*3 = 0.1*ms*^*-1*^, *and k*4 = 0.001*ms*^*-1*^.
Potassium leak current *I*_KL_	
	*M* = 0; *N* = 0;
	*g*KL = 0.03*mS*/*cm*^*2*^;
**For RE cells**	
Fast sodium *I*_Na_ and fast potassium current *I*_K_	
	RE cells have the same *I*_Na_ and *I*_K_ current dynamics as TC cells.
Low threshold calcium current $I_{\underline{T}}$	
	*M* = 2; *H* = 1;
	*m*∞ = 1/(1 + exp(−(*V* + 52)/7.4));
	τm = (3 + 1/(exp((*V* + 27)/10) + exp(−(*V* + 102)/15)))/6.8986;
	*h*∞ = 1/(1 + exp((*V* + 80)/5));
	τh = (85 + 1/(exp((*V* + 48)/4) + exp(−(*V* + 407)/50)))/3.7372;
Potassium leak current *I*_KL_	
	*M* = 0; *N* = 0;
	*g*KL = 0.03*mS*/*cm*^*2*^;

**Table 2:** Model parameters, their values, and descriptions.

Parameter name	Value	Description
**Cortical cells, PY and IN (soma)**		
*g*_Na_	2000 mS/cm^2^ (PY; IN)	Maximal sodium conductance
*g*_K_	200 mS/cm^2^ (PY; IN)	Maximal potassium conductance
*g*_Na(p)_	15 mS/cm^2^ (PY)	Maximal persistent sodium
**Cortical cells, PY and IN (dendrite)**		
*g*_Na_	1.3 mS/cm^2^ (PY; IN)	Maximal sodium conductance
*g*_KL_	0.0033 mS/cm^2^ (PY; IN)	Potassium leakage conductance
*E*_LK_	-95 mV (PY; IN)	Potassium leakage reversal
*g*_L_	0.033 mS/cm^2^ (PY; IN)	Leakage conductance
*E*_L_	-68 mV (PY; IN)	Leakage reversal potential
*g*_Na(p)_	2.5 mS/cm^2^ (PY)	Maximal persistent sodium conductance
*g*_HVA_	0.01 mS/cm^2^ (PY; IN)	Maximal high-threshold Ca^2+^ conductance
*g*_KCa_	0.4 mS/cm^2^ (PY; IN)	Slow Ca^2+^ dependent K^+^ conductance
*g*_Km_	0.014 mS/cm^2^ (PY); 0.03 mS/cm^2^ (IN)	Slow voltage-dependent non-inactivating K^+^ conductance
**Thalamic cells, TC and RE**		
*C*_m_	1 μF/cm^2^	Membrane capacitance
*g*_Na_	90 mS/cm^2^ (TC); 100 mS/cm^2^ (RE)	Maximal sodium conductance
*g*_K_	10 mS/cm^2^ (RE); 10 mS/cm^2^ (TC)	Maximal potassium conductance
*g*_KL_	0.03 mS/cm^2^ (TC); 0.03 mS/cm^2^ (RE)	Potassium leakage conductance
*E*_KL_	-95 mV (TC; RE)	Potassium leakage reversal potential
*g*_L_	0.028 mS/cm^2^ (TC); 0.08 mS/cm^2^ (RE)	Leakage conductance
*E*_L_	-70 mV (TC); -77 mV (RE)	Leakage reversal potential
*g*_T_	1.1 mS/cm^2^ (TC); 2 mS/cm^2^ (RE)	Low-threshold Ca^2+^ conductance
*g*_h_	0.011 mS/cm^2^ (TC)	Hyperpolarization-activated cation conductance

**Table 3:** Synaptic receptors, conductance, and their connecting radii.

Source to target cell	Receptor	Synaptic conductance (μS)	Connecting radius
**Intracortical connections**			
PY→ PY	AMPA	.025	11
PY→PY	NMDA	.0019	11
PY→IN	AMPA	.055	3
PY→IN	NMDA	.001	3
IN→PY	GABA_A_	.055	11
**Intra-thalamic connections**			
TC→RE	AMPA	.1	17
RE→TC	GABA_A_	.05	17
RE→TC	GABA_B_	.02	17
RE→RE	GABA_A_	.05	11
**Thalamocortical connections**			
TC→PY	AMPA	.01	21
TC→IN	AMPA	.01	5
**Cortico-thalamic connections**			
PY→TC	AMPA	.003	21
PY→RE	AMPA	.0015	17

### Cortical intrinsic currents

The PYs and INs of the cortex consist each of a two-compartment (axosomatic and dendritic compartment) model as initially proposed by Mainen and Sejnowski [41] based on Hodgkin-Huxley kinetics [35].


$$\begin{array}{ll} C_{m}\frac{dV_{D}}{dt} & =\;-g_{L}\left(V_{D}-\;E_{L}\right)-\;g_{SD}\left(V_{D}-\;E_{S}\right)-\;I_{D}^{int}-I^{syn},\\ I_{S}^{int} & =\;-\;g_{DS}\left(V_{S}-\;E_{D}\right), \end{array}$$ (1)

In Eq 1, C_*m*_ and g_*L*_ are the membrane capacitance and leakage conductance of the dendritic compartment, respectively. E_*L*_ is the reversal potential, V_*D*_ the dendric and V_*S*_ is the axosomatic compartment membrane potential. g_*SD*_ and g_*DS*_ are the conductance between the axosomatic and dendritic compartments. $I_{D}^{int}$ is the sum of active dendritic and $I_{S}^{int}$ is the sum of active axosomatic currents and *I*^*syn*^ is the sum of synaptic currents. $I_{D}^{int}$ and $I_{S}^{int}$ are the sums of the following intrinsic currents:

$$I_{D}^{int}=I_{Na}+I_{Na\left(p\right)}+I_{LK}+I_{HAV}+I_{Kca}+I_{Km\;},$$

$$I_{S}^{int}=I_{Na}+\;I_{Na(p)}+\;I_{K}.$$

In $I_{D}^{int}$, *I*_*Na*_ is the fast sodium current, *I*_*Na(p)*_ the persistent sodium current, *I*_*LK*_ the potassium leak current, *I*_*HAV*_ the high-threshold calcium current, *I*_*Kca*_ the slow calcium-dependent potassium current, and *I*_*KM*_ the slow voltage-dependent non-inactivating potassium current. Similarly, in $I_{S}^{int}$, *I*_*Na*_ is the fast sodium current, *I*_*Na(p)*_the persistent sodium current, and *I*_*K*_ the delayed rectifier potassium current. PY and IN cells have the same intrinsic currents with the exception of the *I*_*Na(p)*_ current, which is only included in PY cells. The ratio of dendritic to somatic area in PY cells was set to *ρ* = 165, and for IN cells, *ρ* = 50. All individual voltage-dependent currents *I*_*c*_ were simulated in the same fashion as follows:

$$I_{c}=g_{c}m^{M}h^{N}\left(V-E_{c}\right),$$

where *g*_c_ is the maximum conductance, *m* is an activation gating variable, *M* is the number of activation gates, *h* is an inactivation gating variable, and *N* is the number of inactivation gates. *V* is the corresponding compartment voltage and *E*_*c*_ is the reversal potential. Similarly, the dynamics of all gating variables were solved with similar equations as follows:

$$\frac{dy}{dt}=-\frac{x-x_{\infty }}{\tau_{x}}$$

$$\tau_{x}=(1/(\alpha_{x}+\beta_{x}))/Q_{T}$$

$$x_{\infty }=\alpha_{x}/\left(\alpha_{x}+\beta_{x}\right),$$

where x is a gating variable, *x* = *m* or *h*, *Q*_*T*_ = 2.9529, which is a temperature-related term. *α*_*x*_ and *β*_*x*_ are voltage-dependent transition rates. All individual intrinsic currents are described in Table 1 and their units and parametric values are described in Table 2.

### Thalamic intrinsic currents

The thalamic network for sleep spindles also consists of two types of cells: thalamocortical/relay (TC) and reticular (RE) cells. Both cells are modeled based on a single compartment (somatic compartment) with the same voltage-dependent and calcium-dependent current dynamics, as expressed by Hodgkin-Huxley kinetics schemes [35].

$$C_{m}\frac{dV}{dt}=-g_{L}\left(V-\;E_{L}\right)-\;I^{int}-I^{syn},$$ (2)

where *C*_*m*_ is the membrane capacitance, *g*_*L*_ the leakage conductance, *E*_*L*_ the reversal potential, and *V* the voltage of the compartment. *I*^*syn*^ denotes the sum of the synaptic currents and similarly, *I*^*int*^ the sum of the active intrinsic currents. The sum of these active currents for TC, $I_{TC}^{int}$ and RE, $I_{RE}^{int}$ are described as:

$$I_{TC}^{int}=I_{Na}+I_{k}+I_{KL}+I_{h}+I_{T,}$$

$$I_{RE}^{int}=I_{Na}+I_{K}+I_{KL}+I_{T}.$$

In $I_{TC}^{int}$, *I*_*Na*_ is the fast sodium current, *I*_*K*_ the fast potassium current [42], *I*_*LK*_ the potassium leak current, *I*_*T*_ the low-threshold calcium current [39], and *I*_*h*_ the hyperpolarization-activated cation current [43]. In $I_{RE}^{int}$, *I*_*Na*_ is the fast sodium current, *I*_*K*_ the fast potassium current [42], *I*_*LK*_ the potassium leak current, and *I*_*T*_ the low-threshold calcium current [40]. The potassium leak current is *I*_*KL*_ = *g*_*KL*_(*V-E*_*KL*_) in both TC and RE cells where *g*_*KL*_ is the potassium leak conductance and *E*_*KL*_ is the potassium reversal potential (*E*_*KL*_ = -95 mV). All individual voltage-dependent currents were simulated in the same fashion as the cortical intrinsic currents, described in Table 1. Their units and parametric values and synaptic current quantities are given in Tables 2 and 3.

### Synaptic currents

In our model, four types of synaptic currents *I*_syn_ were used, with three of them (AMPA, GABA_A_, and NMDA) modeled by a first-order activation scheme [44, 45] and the fourth, GABA_B_, modeled by a higher-order activation scheme. Accordingly, these synaptic currents are given by:

$$I_{syn}=g_{syn}\left[O\right]f\left(V\right)\left(V-E_{syn}\right),$$ (3)

where *g*_*syn*_ is the maximal synaptic conductance, [*O*] is the fraction of open channels and *E*_*syn*_ is the synaptic reversal potential. For AMPA and NMDA, *E*_*syn*_ = 0 mV, whereas for GABA_A_, *E*_*syn*_ = -70 mV. For AMPA and GABA_A_, *f*(*V*) = 1; for the NMDA receptor, the voltage-dependent sigmoidal function *f*(*V*) = 1/(1+exp(−(*V*−*V*_*th*_)/σ)) was used [42, 44], where *σ* = 12.5 mV and *V*_*th*_ = -25 mV. The fraction of open channel [*O*] was computed by the following equation:

$$d\left[O\right]/dt=\alpha \left(1-\left[O\right]\right)\left[T\right]-\beta [O],$$

$$\left[T\right]=A\theta \left(t_{0}+\;t_{max}-\;t\right)\theta \left(t\;-{\;t}_{0}\right),$$

where *t*_*0*_ is the time for receptor activation and *θ*(*x*) is the Heaviside function [46]. The duration and amplitude parameters for the neurotransmitter pulse were *t*_*max*_ = .03 ms and A = .5. The rate constants for AMPA were *α* = 1.1 ms and *β* = 0.19 ms; for GABA_A_ *α* = 10.5 ms and *β* = 0.166 ms; and for NMDA *α* = 1 ms and *β* = 0.0067 ms. Intracortical currents were modified by multiplying the short-term depression term “*D”* [47, 48] with the maximal synaptic conductance in Eq 3 for AMPA and GABA_A_ receptors.

$$I_{syn}={Dg}_{syn}\left[O\right]f\left(V\right)\left(V-E_{syn}\right),$$

where D is the amount of available synaptic resources, calculated by the following scheme:

$$D_{n+1}=1-\left(1-D_{n}\left(1-U\right)\right)\text{exp}\left(-\frac{\Delta t}{\tau }\right),$$

where the synaptic resources time recovery was *τ* = 700 ms, the interval between *nth* and (*n+1*) *Δt*, and the fraction of resources used for each action potential *U* (for AMPA, *U* = 0.07; for GABA_A_, *U* = 0.073).

The fourth synaptic current, GABA_B_, was computed by a higher-ordered activation scheme that involved the potassium channel activation by a G-protein [49].

$$I_{GABAB}=g_{GABAB}({\left[G\right]}^{4}/({\left[G\right]}^{4}\;+\;K))/(V-\;E_{K})$$

$$d\left[R\right]/dt=K_{1}\left(1-\left[R\right]\right)\left[T\right]-K_{2}$$

$$d\left[G\right]/dt=K_{3}\left[R\right]-\;K_{4}\left[G\right],$$

where [G] is the G-protein concentration, [R] the fraction of activated receptors, and the potassium reversal potential *E*_*K*_ = -95 mV. K_1_ = 0.052 m_M_^-1^ms^-1^, K_2_ = 0.0013 ms^-1^, K_3_ = 0.098 ms^-1^, and K_4_ = 100 μ_M_^4^ were the rate constants. The maximal synaptic conductance for each synapse is described in Table 3.

### Network geometry

The network model is comprised of four one-dimensional layers of neurons (Fig 1). Each layer of cells has N neurons, (N = 40) except the PY neuron layer, which has 5N neurons (200 neurons) [50]. The first and second cortical layers of PY and IN neurons initiate SOs. The third and fourth layers are thalamic layers responsible for initiating sleep spindles. The radii of synaptic connections between different layers are described in Table 3. Each SO cycle is initiated by miniature excitatory postsynaptic potential (EPSP) currents simulated by activation of AMPA receptors of all interconnected PY-PY and PY-IN cells. Poisson noise input is implemented by setting NetStim.noise of the NEURON simulator to 1.

### Stimulation protocols

**Fig 2:**
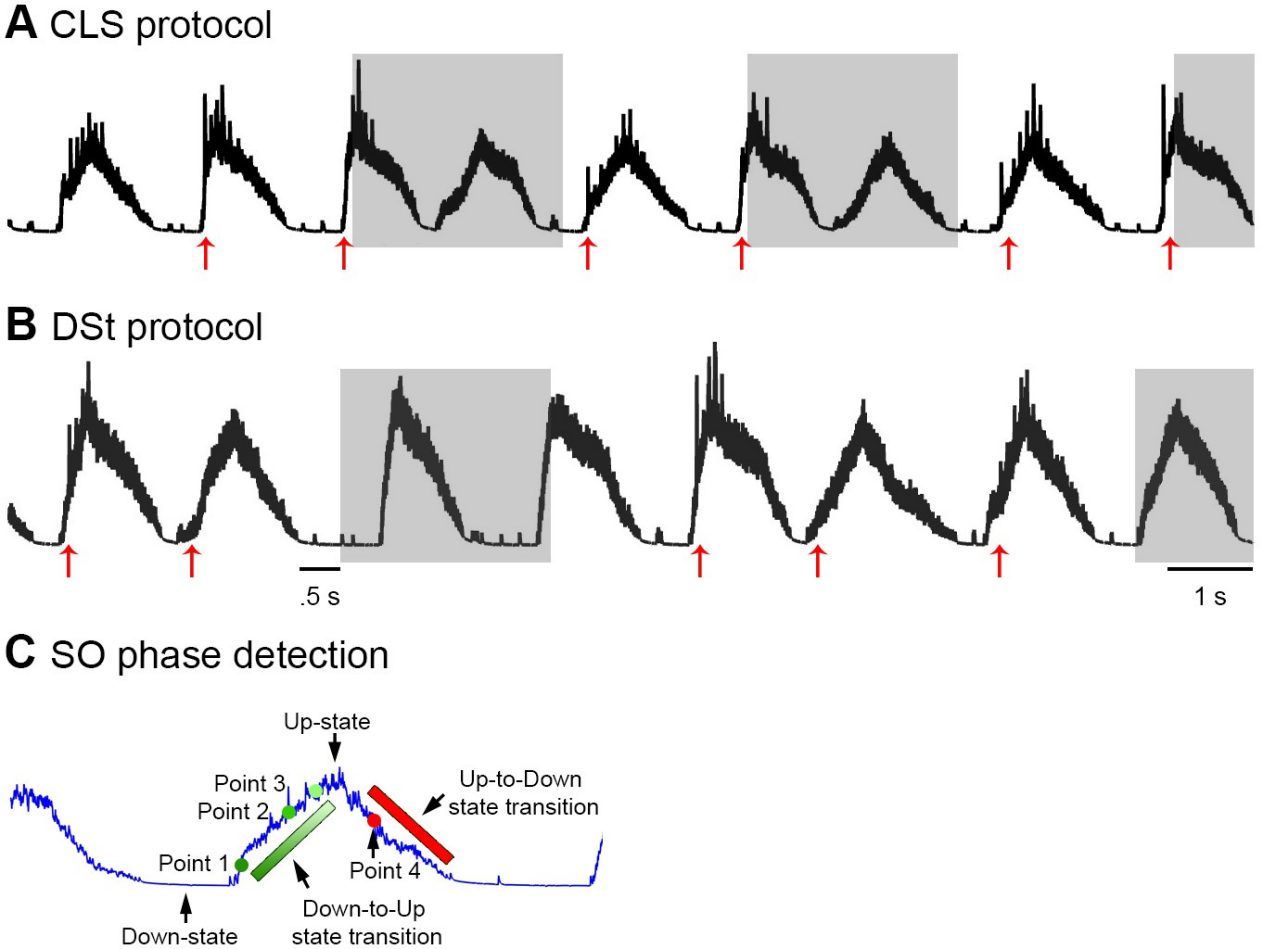
Closed loop stimulation (CLS) and driving stimulation (DSt) protocols. ***A***, In this CLS protocol, stimulation cues (red arrows) were applied immediately after the begin of the SO Down-to-Up-state transition of two successive SOs. After the second cue, SO detection was paused for 2.5 seconds (grey-shaded). ***B***, In this driving stimulation (DSt) protocol, stimulation cues were similarly delivered immediately after the begin of the SO Down-to-Up-state transition. Stimulation was continued for each successive SO as long as the Down-state duration was shorter than 0.5 seconds. ***C***, Different SO states and stimulation points. The dark green circle is the stimulation point immediately after the begin of the Down-to-Up-state transition (“Point 1”, SO detection point), the light green circles present the stimulation points 120 ms (“Point 2”) and 180 ms (“Point 3”) after SO detection, respectively. The red circle is the stimulation point during the Up-to-Down-state transition called “Point 4”. The green and red bars present the SO Down-to-Up and Up-to-Down-state transitions, respectively. The black arrows point to different SO states.

Since the intent of this model is to mimic auditory closed loop stimulation, we introduced a sensory neuron (SN), which sends a sensory cue to the thalamocortical (TC) layer of the thalamic network via AMPA receptors. Following an animal study performed on guinea pigs [51], we attempted to replicate the excitatory postsynaptic potential (EPSP) response to acoustic stimuli. The model also implements a slow oscillation detection algorithm to precisely monitor and detect the different SO states. To detect SO Down-states the algorithm monitors the activity of individual PY cells. If PY cells are silent for > 100 ms the algorithm starts counting the number of spontaneously occurring PY spikes (action potentials). When the number of these spikes reaches 75, the algorithm defines this time point as the end of the Down-state like period and development of the Down-to-Up- state transition (Fig 2C, dark green circle). The stimulation cue is delivered to the TC layer (via SN) relative to this transitory time period (Fig 2). The sensory cue has a duration of 20 ms. We use the term Down-state like period for the model, because it is defined solely by the absence of any spikes. For simplicity we use only “Down-state” in the following.

In this study, CLS, sCLS, and DSt protocols were implemented and their effects on sleep spindle activity were investigated. In CLS, the stimulation cue was applied at the same phase of two successive SOs (Fig 2A, red vertical arrows). After the second of these two cues, the SO detection was paused for 2.5 seconds (Fig 2A, grey shaded area) and subsequently resumed, detecting SOs for the next two clicks and so on. The sCLS protocol has the same procedure as CLS, except here, SO detection was paused for 2.5 seconds after applying a single SO stimulation cue. In the driving stimulation (DSt) protocol, the stimulation cue was applied at every proceeding SO (Fig 2B) as long as the silent Down-state period was shorter than 0.5 second (Fig 2B time mentioned before the first grey shaded area). If the Down-state period was longer than 0.5 s SO detection paused for 2.5 seconds (grey shaded area).

To define different states of SOs, the SO is divided into four states (Fig 2C). The first phase is the Down-state when the entire cortical network goes into a silent state. The second state is the Down-to-Up-state transition commencing when the cortical network starts depolarizing (Fig 2C, green bar). The third is the Up-state when the amplitude of cortical LFP reaches its peak, and the last state is the Up-to-Down-state transition when the amplitude of cortical LFP starts dropping (Fig 2C, red bar) back to a Down-state. In the present study, stimulation cues were topographically applied during multiple time points, but we limit the discussion to four time points as shown in Fig 2C (small colored circles). The first stimulation point “Point 1” was at the commencing of the Down-to-Up-state transition, Fig 2C, dark green circle). When stimulation cues were applied with a time delay of 120 ms and 180 ms (Fig 2C, light green circles) after the commencing of the Down-to-Up state, the terms “Point 2” and “Point 3” were used respectively. For the last stimulation point “Point 4” stimulation cues were applied during the transition from the Up to Down-state (Fig 2C, red circle).

### Computational environment

The model was simulated in the NEURON simulation environment [52] and run on a MacBook Air 2022. For data analysis, MATLAB (R2022b) and the eeglab tool were used.

## Results

We presented a conductance-based thalamocortical model for NREM sleep and its application to simulate closed loop stimulation protocols. Our NREM sleep model exhibits sleep spindles along with SOs: The main network comprises four layers of cells (Fig 1), two of which are cortical layers of PY and IN cells, correspondingly, for SO generation, and two are thalamic layers of TC and RE cells that generate the sleep spindles.

### Results of control simulations

**Fig 3:**
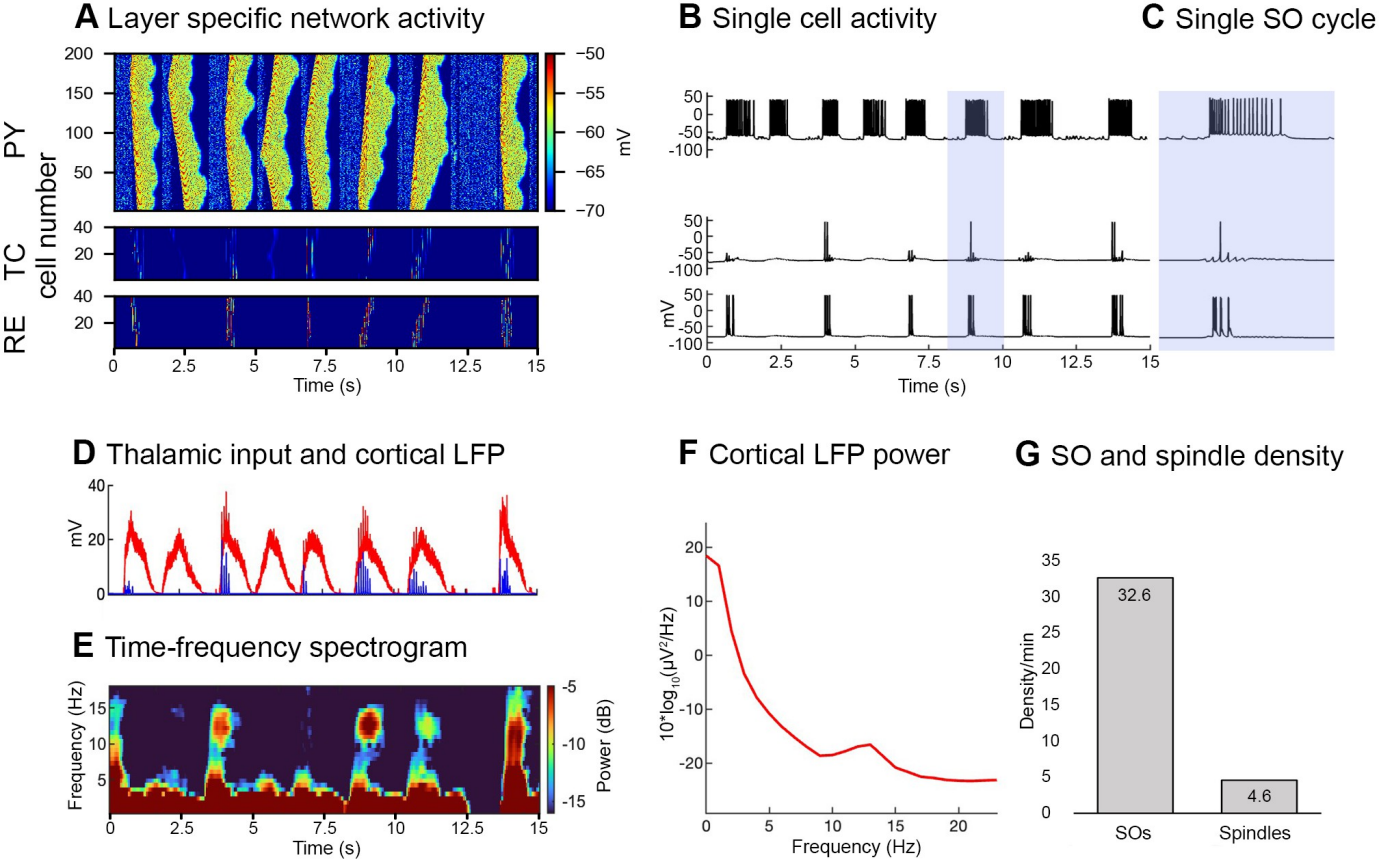
Layer specific network activity. ***A***, Time-space raster plots exhibit the simultaneous activity of pyramidal (PY) and thalamic layers (200 PY cells, 40 cells in each thalamic layer) for a random 15 sec time interval. The membrane potential of each cell is color coded. TC and RE represent thalamocortical and reticular cells of the thalamic network. ***B***, Corresponding single-cell activity within each neuron layer. ***C***, Zoomed single-cell activity of (blue shaded) one slow oscillation (SO) cycle. ***D***, Cortical LFP (red), calculated as the sum of postsynaptic currents (AMPA, GABA_A_ NMDA) of pyramidal (PY) cells. Thalamic activity (AMPA current; blue) occurs at the SO Down-to-Up-state transition of. ***E***, Time-frequency spectrogram of cortical LFP revealing SO and sleep spindle activity (calculated by 1 sec moving Fourier transform window; range, 0.1–18 Hz). ***F***, Power spectrum density (PSD) of the cortical LFP, exhibiting a peak in the sleep spindle (10–16 Hz) frequency band. ***G***, The average number of SOs and spindles per minute.

In Control simulations, the default results of the model without applying stimulation cues are obtained (Fig 3) and later compared with the stimulation results. Activity within the thalamocortical network is initiated by applying a mini synaptic current to the cortical layers during the “Down-state”. Once the SO cycle is initiated, the mini synaptic current is removed from the cortical layers. During the SO Down-state, the mini synaptic current activates the persistent sodium current of PY neurons and, consequently, these PY neurons depolarize and reach their firing threshold. As one or more PY neurons produce an action potential, they target their neighboring PYs by PY-PY connections and sustain this active/depolarized state for 500–1000 ms because of a the strong PY-PY excitation and persistent sodium current. The calcium-dependent potassium current and progressive synaptic depression terminate this active/depolarized state and bring the cortical network back to the Down-state (See Fig 3A for entire network activity and Fig 3B and 3C for single cell activity). One hundred milliseconds after the SO cycle termination, the mini synaptic current is applied again to initiate the next SO cycle and this process is repeated for each SO cycle.

Activation of the cortical network activates both layers of the thalamic network (TC and RE cells layers). In the thalamic network, the interaction of TC and RE cells produces spindle oscillations with major contributions of the TC hyperpolarization current (I_*h*_) and transient calcium current (I_*T*_) [37]. The thalamic output is sent back to the cortical network by TC cells. The cortical network receives this thalamic feedback 50–100 ms after the initiation of the SO cycle (Fig 3D blue traces). The cortical local field potential (LFP) was calculated as the sum of the postsynaptic currents of PY cells (Fig 3D red traces). In the frequency domain, the thalamic network produced spindle activity within the frequency range ~10–16 Hz, shown in Fig 3E (Time-frequency histogram of Fig 3D) and Fig 3F (power spectrum density (PSD) of cortical LFP across the 5 minutes time period). In simulation, an average of 32.6 SOs and 4.6 spindles/min were observed in the Control simulations (Fig 3G). A wider range of spindle densities can be found in the literature. A major review reported an average [53] spindle density of 2.3 ± 2 min^-1^, with values up to 10 min^-1^ [10] in human. After learning Gais et al, 2002 observed a spindle density of 8.4 ± 1.4 min^-1^ [20].

### Results of CLS simulations

**Fig 4:**
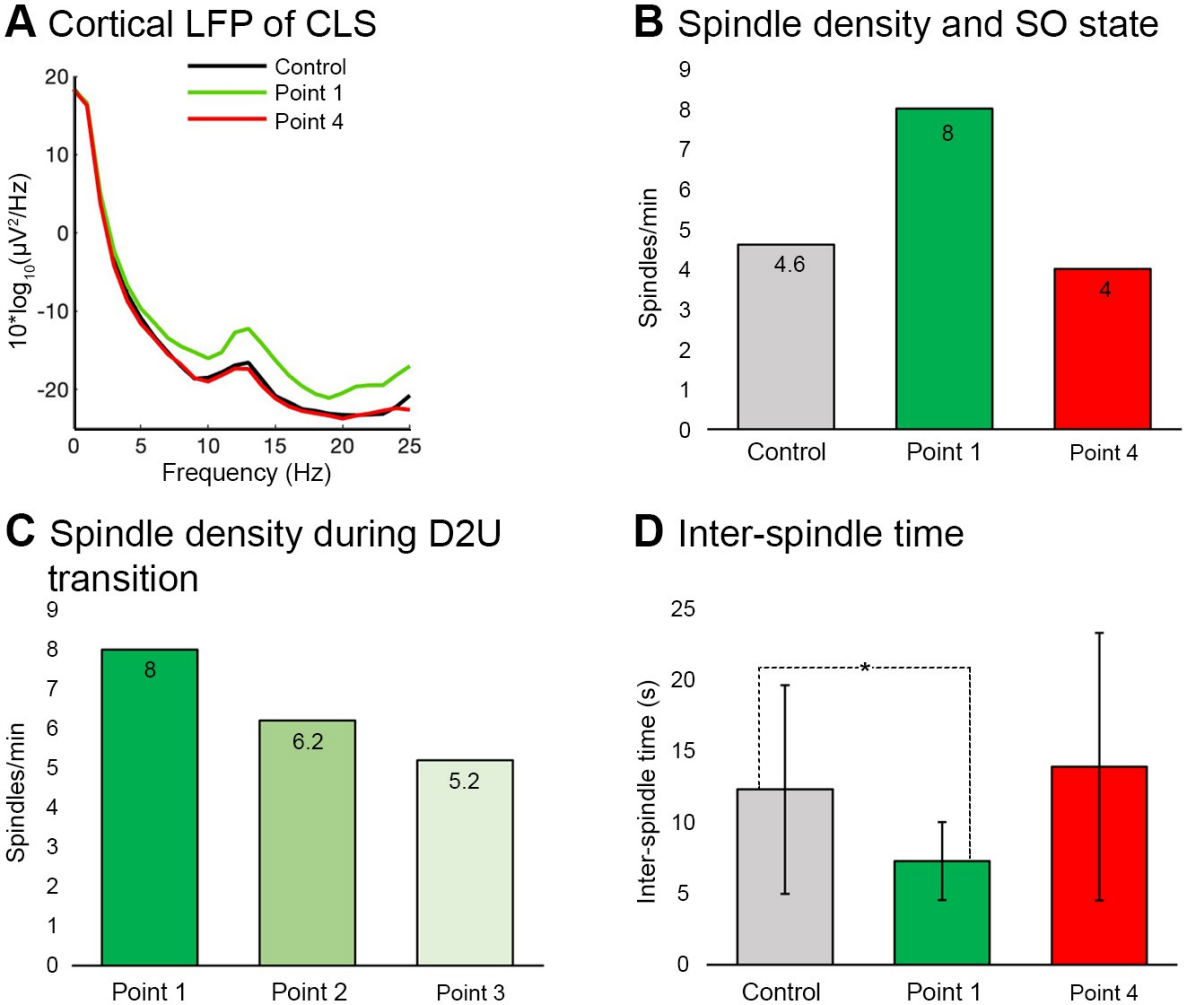
Closed loop stimulation (CLS) simulation results. ***A***, Power spectral density (PSD) of the cortical LFP across a 300 s time period with “Point 1” (green), “Point 4” stimulation (red), and Control (black) simulation. Spindle power was increased compared to Control when the stimulation cue was applied at “Point 1”. Conversely, spindle power was not increased when the cue was applied at “Point 4”. ***B***, Number of spindles in resultant simulations. Average value of spindle density was increased during “Point 1” (green) compared to “Point 4” stimulation (red) and Control simulation (grey). ***C***, Average value of spindle density was decreased when the stimulation cue was applied at both delays after the “Point 1” point. The stimulation cue was less effective with a larger time delay (180 ms) compared to a smaller time delay (120 ms). ***D***, Average inter-spindle time. The average inter-spindle time period was also significantly reduced when stimulation cues were applied at “Point 1” compared to “Point 4” (red) and Control simulation (grey). * P < .005, two sample t-test.

In CLS simulation (Fig 4A) the average value of spindle power was increased when stimulation cues were applied at the “Point 1”. Conversely, the average spindle power was not increased when cues were applied at the “Point 4”. The power spectral density (PSD) of the cortical LFP revealed a strong increase in the average value of sigma band power (10–16 Hz) for stimulations applied at “Point 1” as compared to “Point 4” or the Control simulations (simulations without applying stimulation cue) (Fig 4A). The average value of spindle density for “Point 1” stimulation was also higher than for stimulations at “Point 4” and during the Control simulations (Fig 4B). The average values of spindle density at “Point 1” was also higher than for stimulation at “Point 2” and “Point 3” (Fig 2C). Average values of sigma power in the PSDs of “Point 2” and “Point 3” were also higher than for the Control PSD (not shown in the figure). Interestingly, in a recent human study [54] the stimulation cues were also quite effective during the transition from the Inactive to Active Slow Oscillation (SO) states, as measured in iEEG, resulting in a significant increase in the sigma power. Coming back to our model results, the inter-spindle time which is inverse to spindle density, was significantly lower for “Point 1” than during the Control (7.3 ± 2.7 s vs. 12.3 ± 7.3 s, average ± SD, P < 0.05). Average values at “Point 4”, were higher and reveled a large standard deviation, reflecting spindle events occurring at irregular, large time intervals (14 ± 9.4 s).

In another set of simulations, CLS was applied at “Point 1” with different pause times (2 s, 3 s, and 4 s) between pairs of clicks (see pause time in Fig 2A). In the resultant PSDs of cortical LFP, the sigma band was adequately weaker when the pause time was set to 3 s or 4 s, but the results with 2 s and 2.5 s pause times were approximately the same. In summary, inter-spindle time interval was significantly decreased compared to Control for stimulation applied at the begin of the Down-to-Up-state transition “Point 1”. CLS during Up-to-Down-state transition (Point 4) did not adequately affect spindle activity.

### Results of DSt simulations

**Fig 5:**
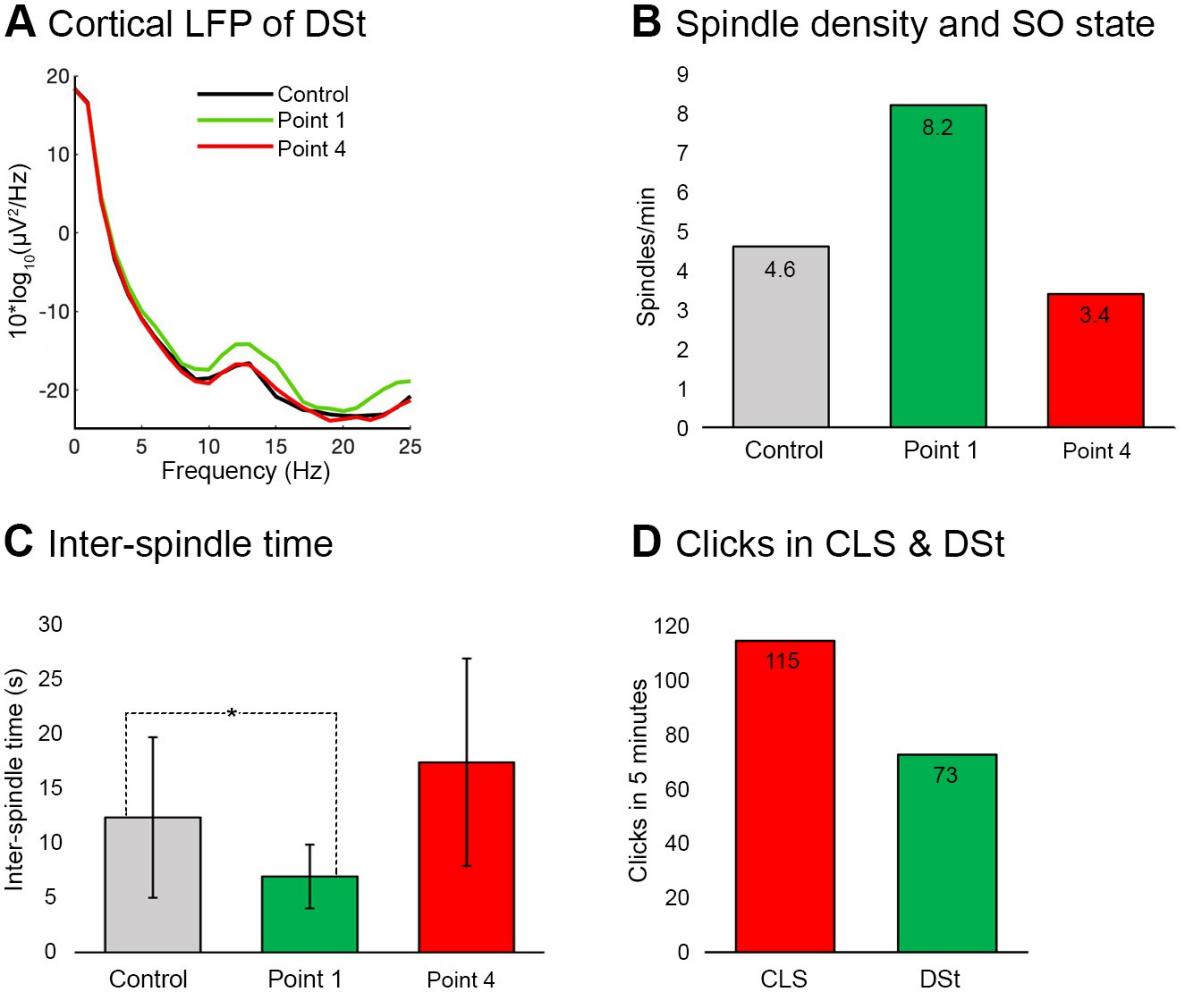
Driving stimulation (DSt) simulation results. ***A***, PSD of the cortical LFP across a 300 s time period with “Point 1” (green), “Point 4” stimulation (red), and Control simulation (black). Spindle power was increased compared to Control when the stimulation cue was applied at “Point 1”. Conversely, this power was not increased when the cue was applied at “Point 4”. ***B***, Number of spindles in resultant simulations. Average value of spindle density was increased when the stimulation cue was applied at “Point 1” (green) compared to “Point 4” stimulation (red) and Control simulation (grey). ***C***, The average inter-spindle time period was also significantly reduced when stimulation cue was applied at “Point 1” compared to “Point 4” stimulation (red) and Control simulation (grey). * P < .005, two sample t-test with. ***D***, Number of clicks (cues) in CLS and DSt simulations across the time period of 300 s. The number of clicks was higher in CLS compared to the DSt simulation.

In the driving stimulation (DSt) protocol, the stimulation cue was applied on every successive SO until its post Down-state time period was less than 0.5 s. As the time period of the Down state exceeded 0.5 s, stimulation was paused for 2.5 s (see Fig 2B). In the DSt protocol average spindle power values in the spindle frequency band were also higher for “Point 1” stimulation than for Point 4 or Control simulation (Fig 5A). Similar to CLS average spindle density at “Point 1” was higher than for “Pont 4” or Control simulation (8.2 vs. 4.6 and 3.4 /min, respectively; Fig 5B). The average number of spindles at “Point 4” was even lower than the average number in the Control simulation. At “Point 1”, the inter-spindle time period was significantly decreased compared to Control simulations, reflecting the increased spindle density at this Point. The spindle events occurred in regular time intervals (7 s, standard deviation; 2.9 s). Conversely, at “Point 4”, spindle events occurred at irregular and large time intervals (17 s, standard deviation; 9.5 s). Interestingly, at “Point 1” the number of clicks in DSt was less than in CLS across 5 minutes of simulation time (73 clicks and 115 clicks respectively, Fig 5D).

### Results of sCLS simulations

**Fig 6:**
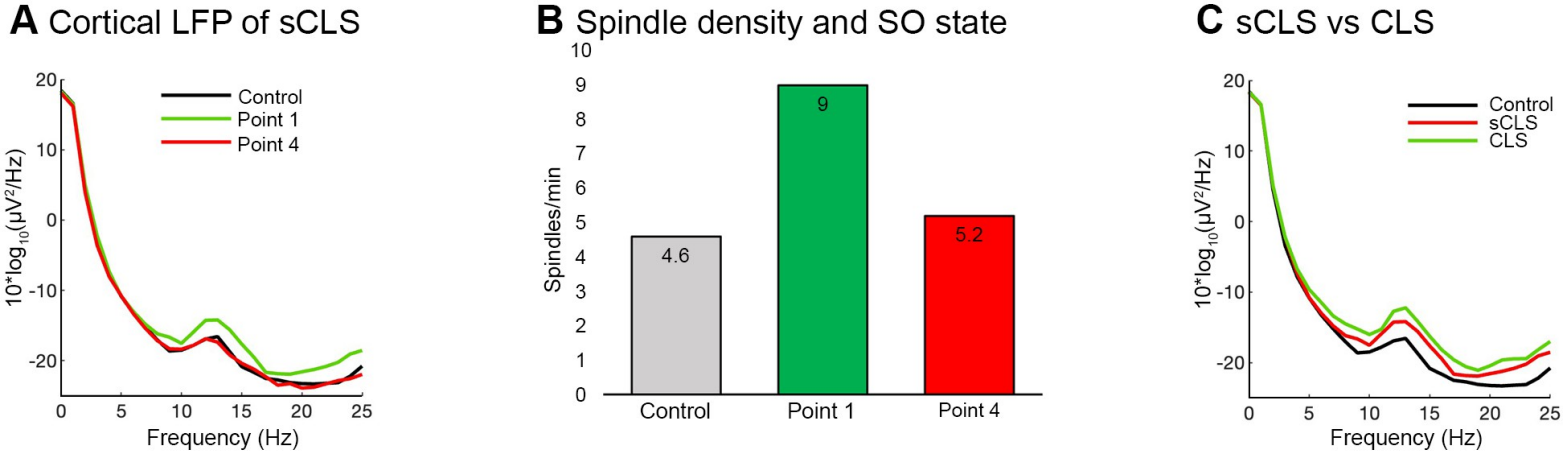
One click closed loop stimulation (sCLS) simulation results. ***A***, PSD of the cortical LFP across a 300 s time period with “Point 1” (green), “Point 4” stimulation (red), and Control simulation (black). Spindle power was increased compared to Control when stimulation cues were applied at “Point 1” like CLS and DSt. ***B***, Average value of spindle density in Control, “Point 1”, and “Point 4” stimulation. ***C***, PSDs of cortical LFP with control simulation, sCLS, and CLS stimulation at “Point 1” stimulation. The sigma power was higher in CLS than in sCLS.

In another set of simulations, one click closed loop stimulation (sCLS) was applied to the TC model. In this stimulation protocol, the stimulation was paused for 2.5 s after each click. In sCLS at “Point 1”, the spindle power was also higher than Control simulations (Fig 6A); particularly, the spindle density was higher (Fig 6B) than the sigma band of corresponding PSD. On the optimal simulation point (“Point 1”) the average sigma band value of sCLS LFP was weaker than the corresponding CLS sigma band, despite its increment compared to Control (Fig 6C). In conclusion, sCLS was effective when the cue was applied at “Point 1”. The delayed cue (particularly on “Point 3”) was less effective in incrementing spindle activity as for the CLS and DSt conditions.

### Potential initiation mechanisms of slow vs. fast spindles

**Fig 7:**
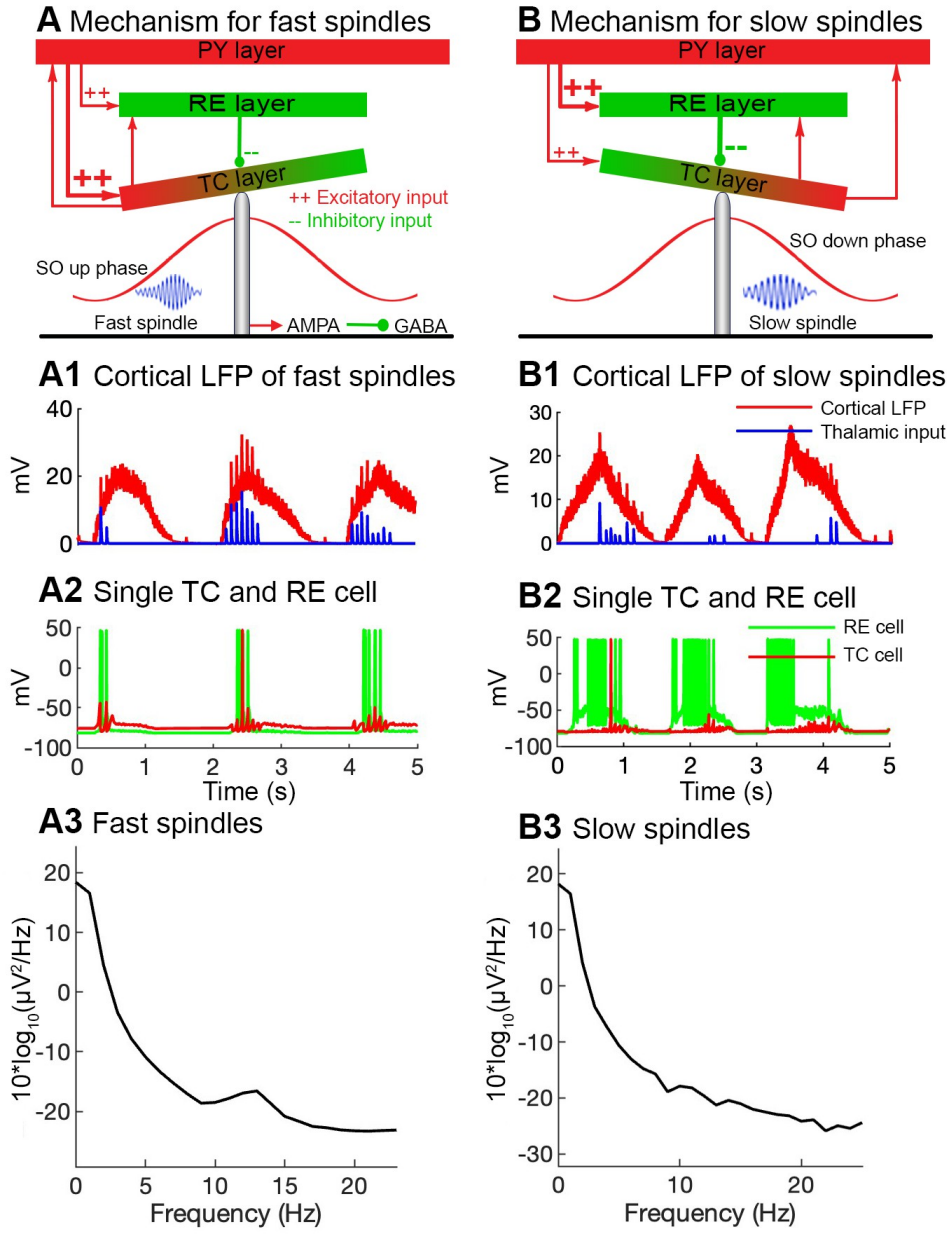
Possible mechanism of the initiation of fast and slow spindles. ***A***, A cartoon diagram of fast spindles’ mechanism. The thalamocortical (TC) layer receives a stronger cortical excitatory input (large red plus sign) from the pyramidal layer, whereas the reticular (RE) layer receives a weaker cortical input. Having a relatively strong excitatory input, the TC layer produces fast thalamic activity which is nested with SOs during the Down-to-Up-state transition (left bottom). ***A1***, The cortical LFP (red) with fast thalamic input (blue) which is nested with cortical LFP during the Down-to-Up-state transition. ***A2***, The corresponding activity of single TC (red) and RE (green) cells. ***A3***, PSD of cortical LFP with fast spindles. ***B***, A cartoon diagram of slow spindles’ mechanism. The RE layer receives a stronger cortical excitatory input (large red plus sign) from the pyramidal layer whereas the TC layer receives a weaker cortical input. Having an excitatory input, the RE layer sends a strong inhibitory input to the TC layer and resultantly, the TC layer produces slow thalamic activity during the Up-to-Down-state transition (left bottom). ***B1***, The cortical LFP (red) with a slow thalamic input (blue) which is nested with cortical LFP during the Up-to-Down-state transition. ***B2***, The corresponding activity of single TC (red) and RE (green) cells. The TC cell activity is observed later (~400 ms) during the SO Up to Down-state ***B3***, PSD of cortical LFP with slow spindles.

In our model, we observed that the shift of cortico-thalamic potential from the TC layer to the RE layer affected spindle density and the phase of their occurrence relative to the SO. Under standard simulation results leading to fast spindle generation, the TC layer received a stronger input than the RE layer from the cortical network (synaptic conductance, PY-TC; 0.003 μS vs. (PY-RE; 0.0015 μS, respectively; (Fig 7A). As a result of this higher cortical input, the TC layer interacted with the RE layer instantly and initiated a fast spindle rhythm which commenced during the SO Down-to-Up-state transition of the cortical LFP (Fig 7A and 7A1). The corresponding single-cell activities of both thalamic layers also simultaneously emerged during the time period of the SO Down-to-Up-state (Fig 7A2).

In another set of simulations, the cortico-thalamic input to the RE layer (PY-RE; 0.0021 μS) was stronger than that to the TC layer (PY-TC; 0.001 μS). In response to this stronger cortical input, activity of the RE layer was increased and sent strong inhibitory feedback to the TC layer (Fig 7B). On fact this inhibitory feedback hyperpolarized and suppressed activity of the TC layer during the Down-to-Up-state transition. This inhibitory feedback gradually decreased, and after approximately ~400 ms the TC layer (now with less RE input became depolarized) interacted with the RE layer and a slow spindle (Fig 7B1 and 7B3) during the Up-to-Down-state transition was initiated. The corresponding single-cell activity of both thalamic layers reveals very strong activity of the RE cells during the Down-to-Up-state transition, while the activity of the TC cell was completely or partially suppressed. Subsequently, (Up-to-Down-state) when RE activity became a bit weaker, the TC cells became depolarized and interacted with RE cells (Fig 7B2), thus producing a slow spindle. Taken together, strong inhibitory input from the RE to the TC layer shifted spindle generation into the Up-to-Down state. The frequency of the generated spindles was also decreased (8–11 Hz) by this inhibition.

In summary, our model showed possible ways to improve the activity of sleep spindles via CLS and DSt protocols and also the role of strong RE activity in the generation of slow spindles our modelling results suggest:

1. Spindle power and density are increased when stimulation cues are applied at the commencement of the SO Down-to-Up-state transition (“Point 1”).
2. The effect of stimulation was decreased when cues were applied with an increased delay compared to “Point 1”.
3. Spindle power and density were not improved over Control when cues were applied during the Up-to-Down-state transition.
4. The sigma power of CLS was stronger than DSt and sCLS during “Point 1” stimulation.
5. A strong inhibitory input of the RE layer to the TC layer shifts the spindle activity to Up-to-Down-state transition (from Down-to-Up state).

## Discussion

In this study, we developed a thalamocortical model for SOs and fast sleep spindles. The SOs are initiated in a two layered (PY and IN layers) cortical network and sleep spindles are initiated by the interaction within two layers (TC and RE Layers) of a thalamic network. Physiologically, fast and slow spindles occur at the Down-to-Up-state transition and at the Up-to-Down-state transition, respectively. In the model, fast spindles are nested with SOs during the first half of the SOs cycle (Down-to-Up-state transition). The stimulation protocols were implemented on the thalamic network. The TC layer received a stimulation cue via AMPA receptors. Two types of stimulation protocols were implemented in the model: CLS and DSt (for details, see *Stimulation protocols* in Methods).

In both CLS and DSt, an increased spindle activity was observed when stimulation cues were applied at the commencing of the Down-to-Up-state transition (Point 1). In addition, the spindle activity gradually decreased when stimulation cues were applied with a time delay from the commencing of the Down-to-Up-state transition. On the other hand, the cues didn’t have a notable effect during the Up-to-Down-state transition (Point 4). The sigma power of CLS results was comparatively higher than DSt results when stimulation cues were applied at “Point 1”, but the spindle density was almost the same in both results. Finally, in another set of simulations, we observed that the slow spindles can be produced by the same thalamic network by the shift of cortico-thalamic potential from the TC layer to the RE layer. A strong inhibitory input from the RE layer to the TC layer shifted the spindle activity to the Up-to-Down-state transition (from the Down-to-Up-state transition) and the frequency of spindles was also reduced (8–11 Hz) by this inhibition.

During slow wave sleep, both cortical and thalamic networks display phase-related synchronous activity in a narrow time window [55]. The sleep spindles and SOs emerge approximately in the same temporal window. Actually, as the cortical network initiates a SO, it also sends feedback to the thalamic network at the same time and ultimately, the thalamic network initiates fast spindles, which are observed a few milliseconds after the initiation of a SO [35].

Although the parameters of our model including cell dynamics matched as close as possible reported physiological activity, as described in the methods, whole-brain responses can be different. As indicated in the methods, the cortical and thalamic network cells were all modelled and compared with the data obtained from experimental results. It was necessary, that these literature sources provided also the corresponding cellular responses with which to compare our results. We remained within the range of intrinsic and synaptic conductances of thalamocortical network cells as documented by Destexhe A. and Sejnowski TJ. [56]. The kinetics of intrinsic currents were estimated from voltage-clamp data in thalamic and cortical cells. In some cases, the relative conductance of each current could also be estimated from published reports. The kinetics of synaptic currents were derived from fits to whole-cell recordings in thalamic, hippocampal, and cortical cells. The simulated responses of single cells under current-clamp were compared to experimental data, an important check for the validity of the parameter values used. A rough estimate for synaptic conductance parameters were obtained from the sizes of EPSPs and IPSPs in various cells following extracellular stimulation. The maximum range of synaptic conductances of thalamocortical connections is documented [56]. Since a variability of parameters given in different studies exists (even with similar experimental conditions), we tuned our parameters within these ranges to obtain a network oscillating in the SO and spindle frequency ranges. Taken together, the synaptic conductance values of our model fall well within the range given in the literature.

In our model when a cortical network initiates a SO, the PY cells start depolarizing and generate action potentials. At the start of the Down-to-Up-state transition, the PY cells exhibit a very strong depolarization, and the cortical LFP rises steeply. Via corticothalamic projection, the thalamic network receives strong synaptic input and this strong input eventually initiates spindles. The resultant thalamic feedback to the cortical network may underlie the fast spindle generation following the SO Down-to-Up-state transition, as also reported in [57]. Our model results suggest that, at the commencing of the Down-to-Up-state transition, the strong corticothalamic input and concurrent stimulation cue synergistically increase spindle activity. On the other hand, the stimulation cues were less effective when the thalamic network received weaker corticothalamic inputs.

In the murine cortex González-Rueda et al. [58] conducted in vivo single-cell recordings, and optogenetically stimulated their presynaptic inputs during SWS. Their results showed that conventional spike timing-dependent plasticity was observed during the Down-states. On the other hand, stimulation during the Up-states was linked to a decrease in EPSP slope. Presynaptic stimulation during the SO Up-state that resulted in postsynaptic spikes protected (but did not enhance) the corresponding connections against synaptic depression. The stimulation protocols of González-Rueda study and our model are nearly similar to each other because in both studies the stimulation cues were applied on a cellular level. Interestingly, in both studies, the stimulation cues were effective approximately at the SO Down-state. A further computational study [59] also observed that, when stimulation cues were applied to the cortical network during the SO Down-state, positive effects were observed on synaptic plasticity. On the other hand, some experimental EEG studies suggested that cues were effective during the SO Up-state [25, 26]. There can be different reasons for these discrepant SO state dependent effects. The foremost reason could be the different system. In the EEG studies there was a delay between the sounding of the cue and the electrophysiological brain response, which was (obviously) absent for the cellular investigations. The other possible reason could be the difference in SO state detection methods. We detected the SO state on the basis of cellular activity whereas Ngo et al. [25, 26] was based on the activity of a neuronal population reflected in the EEG.

In our previous study [34] we presented a thalamocortical model for both fast and slow spindles along with SOs. In that study, we proposed that the slow spindles are possibly initiated in a more hyperpolarized thalamic sub-network, and for their emergence during the second-half SO Up-state, we suggested a synaptic delay of 600 ms time between PY-TC connections. Then we couldn’t find experimental evidence for the 600 ms synaptic delay because of an insufficient number of human studies. In this study, we found an interesting observation about phase shifting without adding a synaptic delay between PY-TC cells. During the first SO phase, a strong RE to TC negative feedback actually hyperpolarized and suppressed the activity of the TC layer. This inhibitory feedback was gradually decreased and after approximately ~400 ms (Fig 7B1), the TC layer became depolarized and interacted with the RE layer and produced slow spindle activity (Fig 7B3) during the second SO phase/ Up-to-Down-state transition.

Functionally, sleep spindles are considered very important for sleep quality [60, 61] neuronal development [53, 62] and synaptic plasticity and memory consolidation. The bursting property of sleep spindles sends a strong synaptic input which eventually triggers synaptic plasticity [63]. This repetitive discrete bursting thalamic activity actually generates robust calcium entry in the dendrites of cortical cells [64]. The entry of calcium might provide an ideal environment to prime synapses for plastic changes [65]. Rosanova and Ulrich [66] showed that sleep spindles induce long-term synaptic changes and also induce Hebbian long-term potentiation in rat somatosensory layer V pyramidal cells.

Taken altogether, our model provides a platform to find the optimal topographical state point of the SO to apply stimulation cues in CLS which eventually improves spindle activity. Furthermore, in the experimental paradigm, the role of reticular network activity needs to be investigated in the occurrence of slow spindles during the SO Up-to-down-state transition. In future work, we will extend this study by including neuronal plasticity into the current model. Furthermore, we will study the role of CLS and increased spindle activity in synaptic plasticity.
